# Efferent Projections of Prokineticin 2 Expressing Neurons in the Mouse Suprachiasmatic Nucleus

**DOI:** 10.1371/journal.pone.0007151

**Published:** 2009-09-28

**Authors:** Chengkang Zhang, Kimberly K. Truong, Qun-Yong Zhou

**Affiliations:** Department of Pharmacology, University of California Irvine, Irvine, California, United States of America; Yale School of Medicine, United States of America

## Abstract

The suprachiasmatic nucleus (SCN) in the hypothalamus is the predominant circadian clock in mammals. To function as a pacemaker, the intrinsic timing signal from the SCN must be transmitted to different brain regions. Prokineticin 2 (PK2) is one of the candidate output molecules from the SCN. In this study, we investigated the efferent projections of PK2-expressing neurons in the SCN through a transgenic reporter approach. Using a bacterial artificial chromosome (BAC) transgenic mouse line, in which the enhanced green fluorescence protein (EGFP) reporter gene expression was driven by the *PK2* promoter, we were able to obtain an efferent projections map from the EGFP-expressing neurons in the SCN. Our data revealed that EGFP-expressing neurons in the SCN, hence representing some of the PK2-expressing neurons, projected to many known SCN target areas, including the ventral lateral septum, medial preoptic area, subparaventricular zone, paraventricular nucleus, dorsomedial hypothalamic nucleus, lateral hypothalamic area and paraventricular thalamic nucleus. The efferent projections of PK2-expressing neurons supported the role of PK2 as an output molecule of the SCN.

## Introduction

The primary mammalian circadian clock resides in the suprachiasmatic nucleus (SCN) of the hypothalamus. The SCN drives the behavioral and physiological circadian rhythms, such as locomotor activity, sleep and wakefulness, feeding, energy metabolism and body temperature [1]–[3]. Dye tracing experiments have revealed that the primary efferent target areas of the SCN are quite limited and predominantly locate in the hypothalamus and the midline thalamus. Primary SCN target areas include lateral septum, bed nucleus of the stria terminalis, subparaventricular zone, paraventricular hypothalamic nucleus, dorsomedial hypothalamic nucleus as well as paraventricular thalamic nucleus [3]–[8]. Efferent projections of the SCN to its target sites are also inferred by the distributions of vasopressin (AVP) and vasoactive intestinal peptide (VIP) positive fibers, which largely overlaps with each other in all known SCN target sites within the hypothalamus [5], [9]. The efferent projections of the mouse SCN correspond to what have been described in hamster and rat [4], [6]–[8]. Both SCN subdivisions are believed to be capable of disseminating circadian information to the thalamus, hypothalamus and basal forebrain [9].

Transplantation of viable embryonic SCN tissues can partially restore the locomotor rhythm in SCN-lesion animals, and the rhythms restored by the transplants display the characteristics of the donor circadian pacemakers rather than those of the hosts [10]–[13]. Most interestingly, neuronal connectivity of the transplants was not established in these studies, suggesting that some humoral factors may be sufficient to transmit the circadian information from the SCN, at least for the locomotor rhythms. However, transplantation of embryonic SCN tissue does not restore the endocrine and other physiological rhythms, underscoring the importance of axonal connections between the SCN and its target sites for the regulation of many other circadian processes [14].

Prokineticin 2 (PK2) is a cysteine-rich secreted protein that exhibits high circadian rhythmic expression in the SCN, and its transcription is tightly controlled by components of the core molecular circadian oscillators [15]–[17]. Recently, genetic studies have revealed that PK2 regulates the circadian rhythms of locomotor activity, sleep and wakefulness, feeding and body temperature [18]–[19]. One receptor for PK2, prokineticin receptor 2 (PKR2), has been shown to be expressed in most primary target areas of the SCN by mRNA *in situ* hybridization [15], [20], as well as ligand binding autoradiography [21]. In addition, targeted null mutation of the mouse *PKR2* gene also disrupts the circadian rhythms, resulting in nearly identical phenotypes as the PK2 mutant mice [21]–[22]. One important perspective to comprehend the functions of PK2 in regulating the circadian system is to explore the characteristics of PK2-expressing neurons in the SCN and their connections in the context of neuronal circuitry. Unfortunately, no antibody against PK2, despite great efforts from multiple groups, was available for immunohistochemical study to date. In the current study, we obtained the projection map of a subset of PK2-expressing neurons in the SCN, utilizing a bacterial artificial chromosome transgenic mouse.

## Results

### Generation of PK2-EGFP transgenic mouse

**Figure 1:**
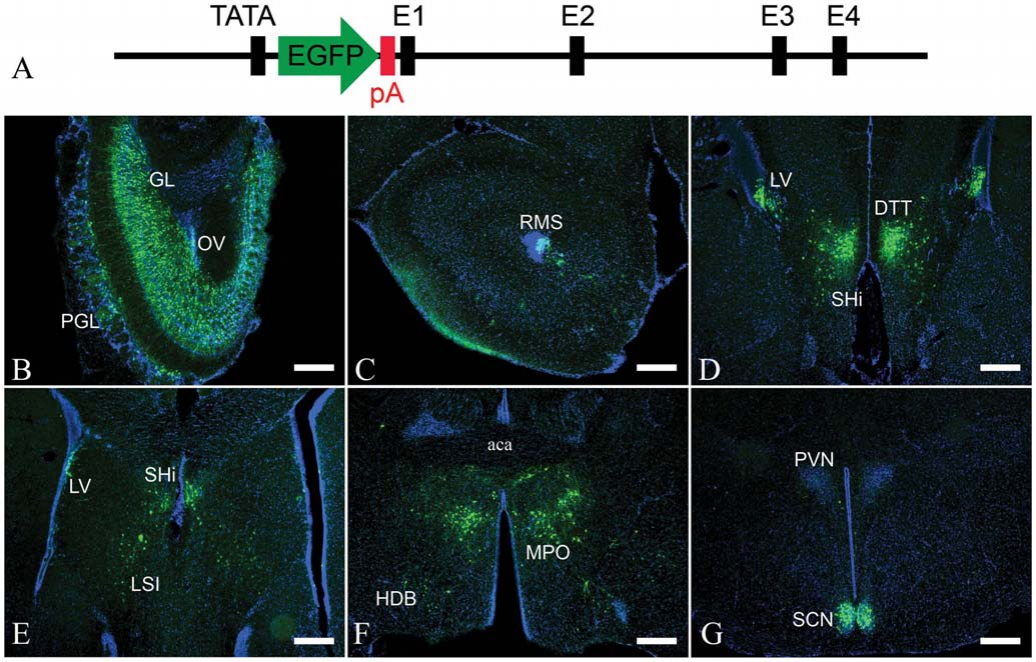
EGFP reporter expression in the brain of PK2-EGFP transgenic mice. A. Schema showing the insertion of EGFP reporter cassette into the *PK2* gene in the BAC clone transgenic construct. TATA, *PK2* gene promoter; pA, polyadenylation site in the EGFP reporter cassette; E1–E4, four exons of *PK2* gene. B–G. Immunofluorescence staining showed EGFP-ir cells in B) the granule layer (GL), periglomerular layer (PGL) and olfactory ventricle (OV) of the olfactory bulb; C) the rostral migration stream (RMS); D) the lateral ventricle (LV), septohippocampal nucleus (SHi) and dorsal tenia tecta (DTT); E) the intermediate lateral septum (LSI), LV and SHi; F) the medial preoptic nucleus (MPO) and horizontal limb of the diagonal band of Broca (HDB); G) the suprachiasmatic nucleus (SCN). The animals were sacrificed at ZT12. Cell nuclei were counter-stained in blue. Scale bar  = 100 µm.

To construct the transgenic mouse line, BAC clone RP23-12A18 was modified to insert an EGFP reporter cassette between the promoter and the first exon of *PK2* gene (Figure 1A). For the transgenic allele, transcription would be presumably driven by the *PK2* promoter and stop after the polyadenylation site in the EGFP reporter cassette, resulting in EGFP expression without the overexpression of PK2. Indeed, we did not detect an increase in *PK2* mRNA expression by *in situ* hybridization in the transgenic mice, compared with wild-type non-transgenic mice (data not shown).

### Distribution of EGFP-expressing cells in the brain

We started by surveying the expression of EGFP reporter in the brains of 8–10 weeks old transgenic mice. Decent direct fluorescence in the cell soma could be seen in discrete areas of the adult brain, such as the olfactory bulb (OB), medial preoptic area (MPO) and SCN (data not shown). Either avidin-biotin based immunohistochemistry or indirect immunofluorescence staining significantly increased the EGFP signal, especially in the fibers. Since immunohistochemistry and immunofluorescence staining resulted in comparable signals, we used the latter to enable multiple signals detection.

As expected, many EGFP-immunostained (EGFP-ir) neurons were seen in the SCN (Figure 1G, see below for details). Besides that, copious EGFP-ir cells were seen in the granule layer (GL) and periglomerular layers (PGL) of the OB, with many cells exhibiting the morphologies of interneurons (Figure 1B). Quite a few EGFP-ir cells were also observed in subventricular layer of the olfactory ventricle (OV), the rostral migration stream (RMS) and the subventricular layer of the lateral ventricle (LV). Most of these EGFP-ir cells looked like migrating neuroprogenitors, although a few appeared glia-like (Figure 1B, C and D). Many neurons in the dorsal tenia tecta (DTT), septohippocampal nucleus (SHi) and intermedial lateral septum (LSI) were also positive for EGFP (Figure 1D and E). The most intense signals for EGFP were seen in the preoptic area, including the anterodorsal preoptic nucleus (ADP) and MPO, while another group of EGFP-ir neurons were also observed in the horizontal limb of the diagonal band of Broca (HDB) (Figure 1F). There were also a handful of EGFP-ir neurons in the arcuate nucleus (Arc). No EGFP-ir cells were seen in the midbrain or the hindbrain (data not shown).

### EGFP-immunostained fibers in most SCN target sites

**Figure 2:**
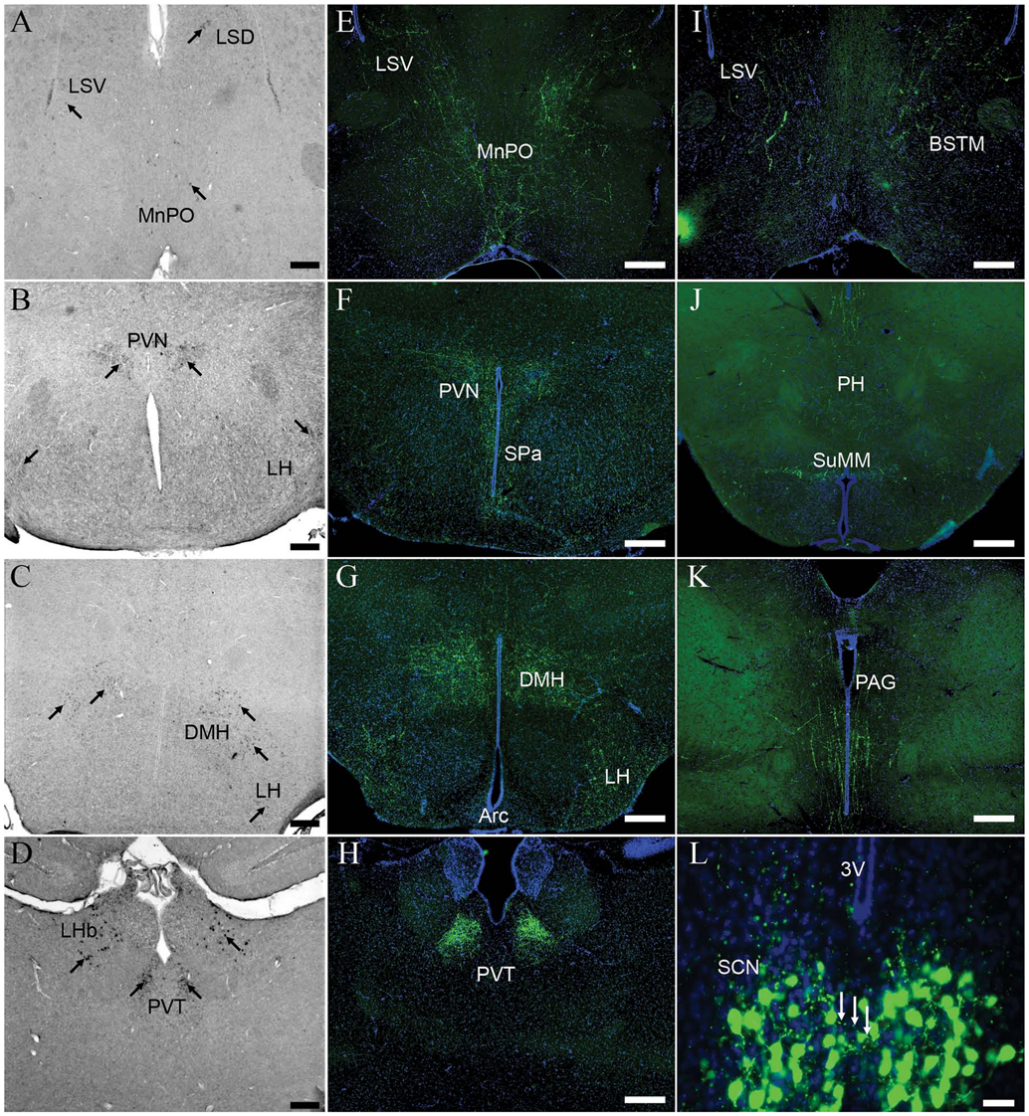
Distribution of EGFP-immunostained fibers in the SCN target areas and complementary expression of PKR2. A–D. Digoxigenin-labeled *in situ* hybridization showed the expression of *PKR2*. Arrows indicated *PKR2*-positive cells. DMH, dorsomedial hypothalamic nucleus; LH, lateral hypothalamic area; LHb, lateral habenular nucleus; LSD, dorsal lateral septum; LSV, ventral lateral septum; MnPO, median optical area; PVN, paraventricular nucleus; PVT, paraventricular thalamic nucleus. Scale bar  =  200 µm. E–K. Immunofluorescence staining showed EGFP-ir fibers in E) the median preoptic area (MnPO) and ventral lateral septum (LSV); F) the subparaventricular zone (SPa) and paraventricular nucleus (PVN); G) the lateral hypothalamic area (LH), dorsomedial hypothalamic nucleus (DMH) and arcuate nucleus (Arc); H) the paraventricular thalamic nucleus (PVT); I) the bed nucleus of the stria terminalis, medial (BSTM) and LSV; J) the posterior hypothalamic area (PH) and medial supramammillary nucleus (SuMM) and K) periaqueductal gray (PAG). Scale bar  =  100 µm. L. A high magnification view of EGFP-ir cells and fibers inside the suprachiasmatic nucleus (SCN). Arrows indicated EGFP-ir fibers that extended from one nucleus into the contralateral nucleus. Scale bar  =  20 µm. 3V, third ventricle. The animals were sacrificed at ZT12. Cell nuclei were counter-stained in blue in E–L.

**Figure 3:**
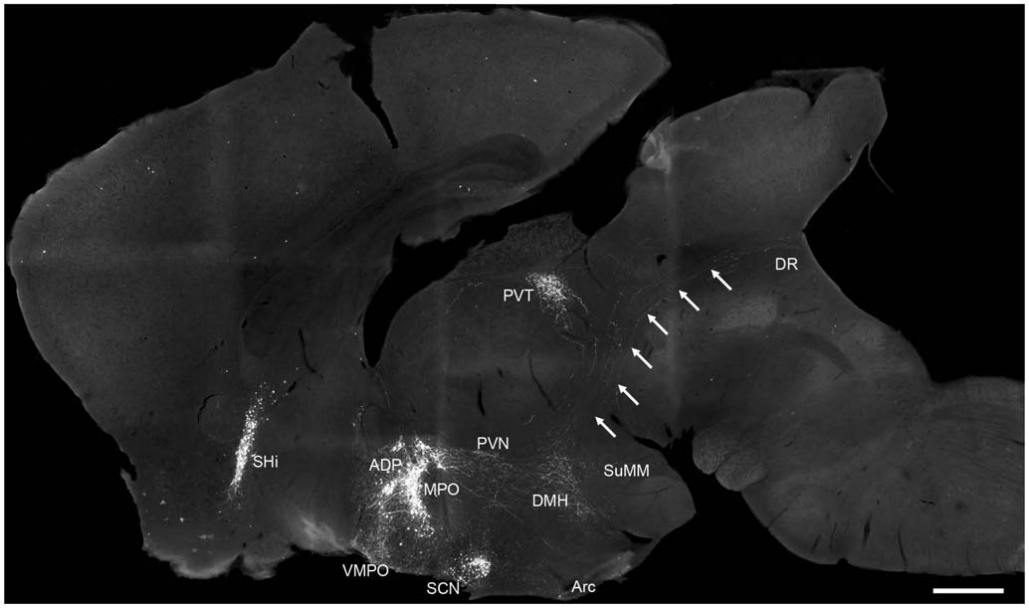
Trajectory of EGFP-immunostained fibers in the hypothalamus, thalamus and midbrain. In a parasagittal plane close to the midline, cells with strong EGFP-ir signals could be seen in the septohippocampal nucleus (SHi), anterodorsal preoptic nucleus (ADP), medial preoptic nucleus (MPO) and the suprachiasmatic nucleus (SCN). Strong EGFP-ir fibers could be seen in the ventromedial preoptic nucleus (VMPO), the preoptic area, paraventricular nucleus (PVN), dorsomedial hypothalamic nucleus (DMH), arcuate nucleus (Arc), medial supramammillary nucleus (SuMM), paraventricular thalamic nucleus (PVT) and dorsal raphe nucleus (DR). The animals were sacrificed at ZT12. Arrows indicated the EGFP-ir fibers coursed into the dorsal raphe nucleus. Scale bar  =  500 µm.

Extensive EGFP-ir fibers were observed in most known SCN target areas in the septum, preoptic area, hypothalamus, thalamus and midbrain of the adult transgenic mouse brain (Figure 2 and Table 1). Dense EGFP-ir fibers could be seen coursing through the median preoptic area (MnPO), with many of them continued dorsally into the medial bed nucleus of the stria terminalis (BSTM) and the ventral lateral septum (LSV) (Figure 2E and 2I). In the hypothalamus, the densest plexus of EGFP-ir fibers from the SCN began just dorsal and caudal to the nucleus, then vertically projected into the ipsilateral subparaventricular zone (SPa) and continued dorsally to a region ventral to the magnocellular part of the posterior paraventricular hypothalamic nucleus (PVN, Figure 2F). Inside the caudal part of SCN, a handful of EGFP-ir fibers could be seen crossing into the contralateral nucleus (Figure 2L). Posterior to the PVN, the dorsomedial hypothalamic nucleus (DMH) and lateral hypothalamic area (LH) received vast innervations of the EGFP-ir fibers. In contrast, there were few EGFP-ir fibers in the ventromedial hypothalamic nucleus (Figure 2G). A few EGFP-ir fibers were also observed in the Arc, posterior hypothalamic area (PH), lateral and medial supramammillary nucleus (SuMM, Figure 2J). In the thalamus, substantial EGFP-ir fibers could be seen extending dorsally and innervating the paraventricular thalamic nucleus (PVT) (Figure 2H). In the midbrain, intensive EGFP-ir fibers were observed throughout the length of the periaqueductal gray (PAG, Figure 2K), many of which probably extended into the dorsal raphe nucleus (DR, see Figure 3 and below for details).

**Table 1:** Distribution of EGFP-immunoreactive neurons and projections in adult mouse brain.

Brain regions	Abbreviation	EGFP+ neurons	PK2 mRNA	EGFP+ fibers	PKR2 mRNA
**Olfactory regions & Ventricles**					
Granule layer	GL	+++	++	+++	+
Periglomerular layer	PGL	++	++	++	−
Olfactory ventricle	OV	+	+	−	++
Lateral olfactory tract	LOT	−	−	++	−
Olfactory tubercle	Tu	−	−	++	−
Rostral migration stream	RMS	+	+	−	++
Subventricular zone of lateral ventricle	SVZ	+	+	+	++
**Septum and Basal ganglia**					
Lateral septum, Dorsal	LSD	−	−	−	+
Lateral septum, Intermediate	LSI	+/++	−	++	+
Lateral septum, Ventral	LSV	+/−	−	+	+
Septohippocampal nucleus	SHi	++	−	++	−
Major island of Calleja	ICjM	+	+	−	−
Dorsal tenia tecta	DTT	++	−	++	−
**Thalamus**					
Paraventricular thalamic nucleus, Anterior	PVA	−	−	+	+++
Paraventricular thalamic nucleus	PVT	−	−	+++	++
Lateral habenular nucleus	LHb	−	−	+/−	++
**Hypothalamus**					
Medial preoptic area	MPA	−	−	+++	−
Lateral preoptic area	LPA	−	−	++	−
Median preoptic area	MnPO	−	−	++	+
Vascular organ of the lamina terminalis	VOLT	−	−	++	−
Bed nucleus of the stria terminalis, Medial	BSTM	−	−	+	+
Medial preoptic nucleus	MPO	+++	+++	+++	−
Anterodorsal preoptic nucleus	ADP	++	++	++	−
Horizontal limb of the diagonal band of Broca	HDB	+	−	+	−
Suprachiasmatic nucleus	SCN	+++	+++	++	+++
Subparaventricular zone	SPa	−	−	+++	+
Paraventricular nucleus	PVN	−	−	+/++	+/++
Dorsomedial hypothalamic nucleus	DMH	−	−	+++	++
Lateral hypothalamic area	LH	−	−	++	+
Arcuate nucleus	Arc	+/−	+/−	+	+
Posterior hypothalamic area	PH	−	−	+	−
Mammillary nucleus, Medial	MM	−	−	+	−
Mammillary nucleus, Lateral	ML	−	−	+	−
Supramammillary nucleus, Medial	SuMM	−	−	+	+
Supramammillary nucleus, Lateral	SuML	−	−	+	+
**Midbrain**					
Periaqueductal gray	PAG	−	−	++	+
Dorsal raphe nucleus	DR	−	−	+	+

It is important to note that many cells in these SCN target areas expressed *PKR2*, a G-protein coupled receptor for PK2, as observed by *in situ* hybridization on adjacent sections (Figure 2A–D). For example, *PKR2* mRNA-expressing cells were seen in the MnPO, LSV and LSD (Figure 2A), BSTM (data not shown), PVN, LH (Figure 2B), DMH (Figure 2C), Arc (data not shown), lateral habenular nucleus (LHb), PVT (Figure 2D) and DR (data not shown), suggesting that these areas were able to receive PK2 signaling from SCN.

Trajectory of the EGFP-ir axons could be evidently viewed in the sagittal plane (Figure 3). A great number of EGFP-ir neurons could be seen in the SCN, MPO and ADP. Dense plexus of EGFP-ir fibers were evident in the preoptic area, PVN, DMH and PVT. Particularly, a heavy bundle of EGFP-ir fibers could be seen curving posteriorly through the PVN and extensively innervating the DMH and posterior hypothalamic area. Many EGFP-ir fibers then exhibited an “S”-shaped trajectory along the boundary between thalamus and midbrain, and could be traced further into the DR (marked by arrows in Figure 3). There was a strong plexus of EGFP-ir fibers innervating the posterior PVT, coursing along the posterior boundary between thalamus and midbrain. A few of EGFP-ir fibers also curved along the anterior thalamus and extended into the PVT (Figure 3).

**Figure 4:**
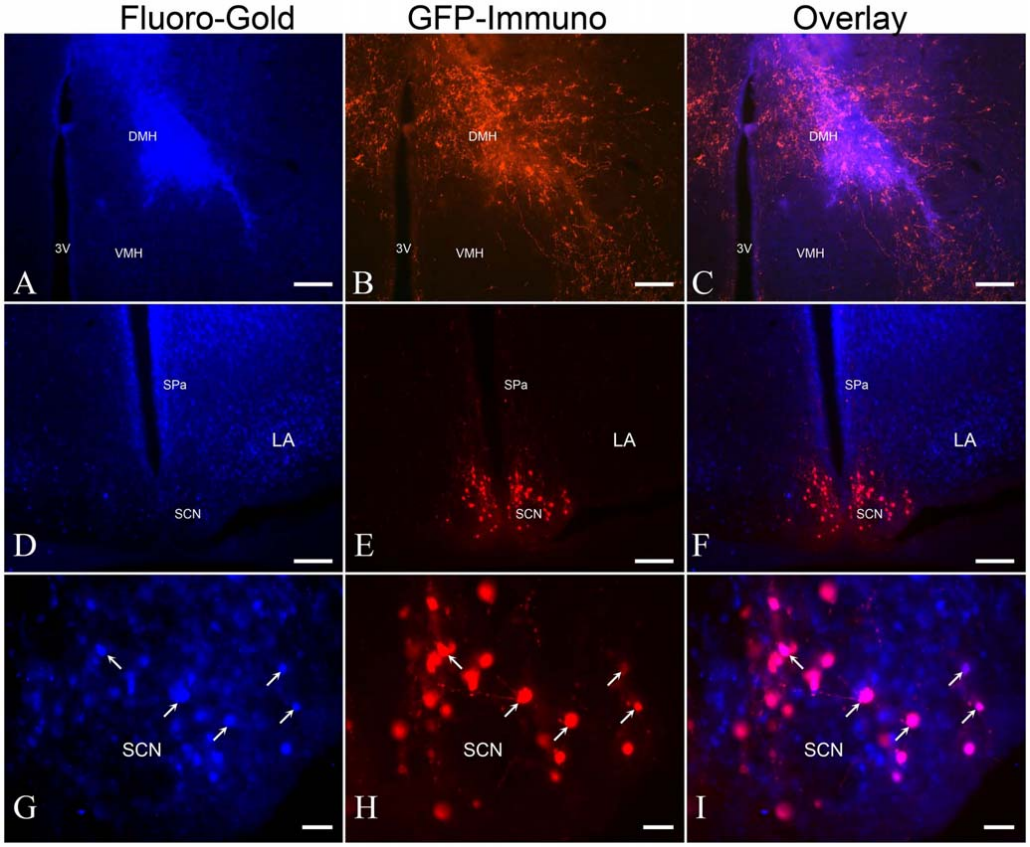
Fluoro-Gold retrograde tracing. A–C. Fluoro-Gold was delivered into the DMH, which exhibited a heavy plexus of EGFP-ir fibers in the adult transgenic mice brain. Scale bar  =  100 µm. D–F. Plenty of Fluoro-Gold labeled cells could be seen in the subparaventricular zone (SPa), lateroanterior hypothalamic nucleus (LA) and the SCN. Scale bar  =  100 µm. G–I. Arrows indicated a few of EGFP-ir cells in the dorsomedial SCN which was retrogradely labeled by Fluoro-Gold. The animals were sacrificed at ZT12. Scale bar  =  20 µm.

We used the retrograde axonal tracer Fluoro-Gold to confirm the origin of EGFP-ir fibers in the DMH, which receives inputs from other regions of the hypothalamus [23]–[24]. In four of twenty-four mice, Fluoro-Gold deposits were restricted primarily within the DMH (Figure 4). We focused our analysis on the distribution of retrogradely labeled neurons in the SCN and the preoptic area. Fluoro-Gold labeled neurons appeared to be widespread in the SCN. Remarkably, quite a few of the EGFP-ir neurons in the dorsomedial part of SCN were labeled by Fluoro-Gold (Figure 4G–I), indicating that these neurons projected into the DMH. In addition, significant numbers of neurons in the SPa and lateroanterior hypothalamic nucleus (LA) were also labeled by Fluoro-Gold (Figure 4D–F), consistent with known projection between these regions and the DMH. Retrogradely labeled neurons were also observed throughout the preoptic area (data not shown), in agreement with previous study on inputs to the DMH in the rat [23].

### EGFP-immunostained neurons represented a subset of PK2 mRNA-expressing neurons in the SCN

**Figure 5:**
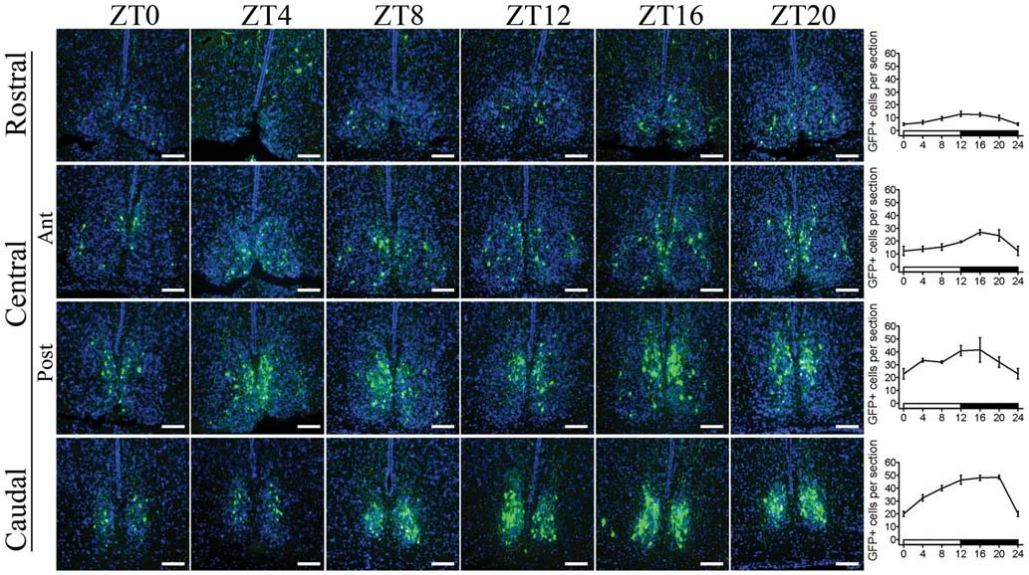
Circadian oscillation of the number of EGFP-positive neurons in the SCN. Comparisons of consecutive coronal sections through the rostral to caudal extent of the SCN at different zeitgeber time (ZT) points showed that more EGFP-positive cells located in the caudal half of the SCN than the rostral part. In both rostral and caudal parts of the SCN, the EGFP signals peaked around ZT12. Scale bar  =  50 µm. Cell nuclei were counter stained in blue.

The expression of *PK2* mRNA in the SCN exhibits profound circadian rhythm [15], [25]. To explore whether the EGFP reporter imitated the daily rhythms of *PK2* mRNA, we characterized the regional and temporal distribution of EGFP-ir cells from the rostral to central (anterior and posterior portions) and caudal quadrants of the SCN during 24 hours (Figure 5). Overall, there were less EGFP-ir cells in the rostral and anterior central quadrants of SCN than in the posterior central and caudal quadrants (20±4 vs. 94±9 EGFP-positive cells on each 14 µm section at ZT12, p<0.001, non-parametric Kruskal-Wallis test, three animals). During 24 hours, the number of EGFP-ir cells in the SCN showed a modest oscillation, declining to its nadir at ZT0 and escalating to its peak around ZT12 in both rostral and caudal halves of the SCN, with the latter showing greater oscillation amplitude (Figure 5. Effect of time: F(6,56) = 42.53, p<0.001; Effect of position: F(3,56) = 219.45, p<0.001; two-way ANOVA).

**Figure 6:**
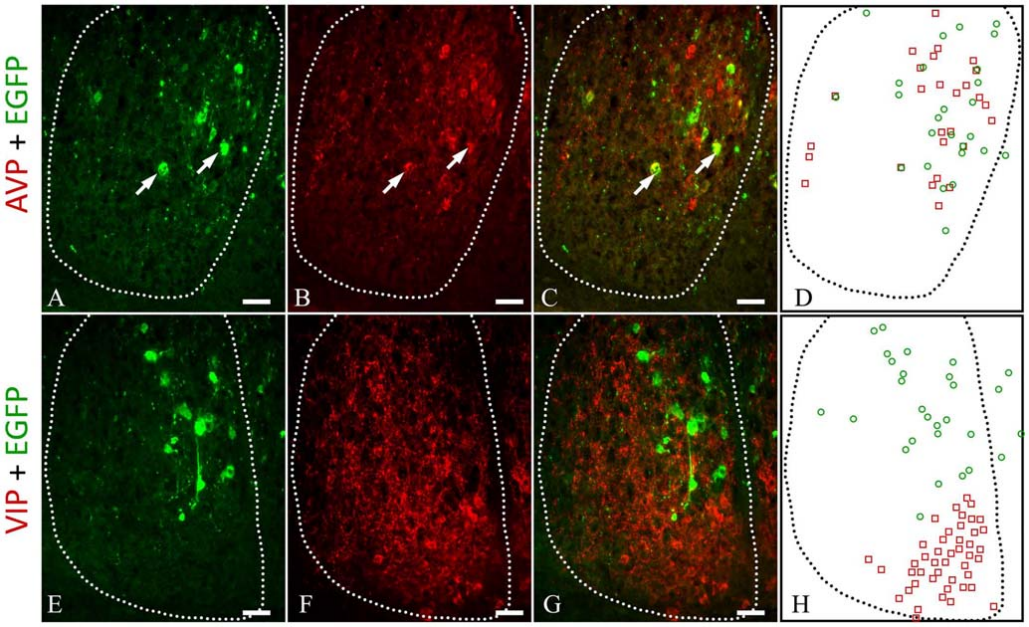
A subset of EGFP-ir cells in the SCN co-expressed vasopressin (AVP). A–C. Double immunostaining against EGFP and AVP in the SCN. Cells that were positive for both EGFP and AVP signals were marked by arrows. D. The localizations of EGFP- and AVP-positive neurons in the SCN were shown schematically. E–G. Double immunostaining of EGFP and vasoactive intestinal peptide (VIP) in the SCN. No neuron was positive for both EGFP and VIP signals. H. The localizations of EGFP- and VIP-positive neurons in the SCN were shown schematically. In all images, the boundary of SCN was indicated by the dotted line. The third ventricle was to the right in all images. The animals were sacrificed at ZT9. Scale bar  =  20 µm.

A closer look at the regional distribution of EGFP-ir neurons inside the SCN showed that the EGFP-ir neurons and processes were limited to the dorsomedial and lateral edges of the nucleus in the rostral and anterior-central compartments, creating the appearance of a shell. In the posterior central portion of the SCN, majority of the EGFP-ir cells were found in the medial and dorsomedial areas. While in the caudal quadrant, EGFP-ir cells dispersed throughout the SCN (Figure 5). As for the phenotype of the EGFP-ir neurons, about 60% of the EGFP-ir neurons in the SCN were also positive for vasopressin (AVP) at ZT12, as shown by double immunostaining with antibodies against AVP and EGFP (Figure 6A–D, EGFP+ cells: 606±74; AVP+ cells: 2574±198; EGFP+/AVP+ cells: 357±32, three animals). On the contrary, no EGFP-ir cell was positive for vasoactive intestinal peptide (VIP) at the same condition (Figure 6E–H).

**Figure 7:**
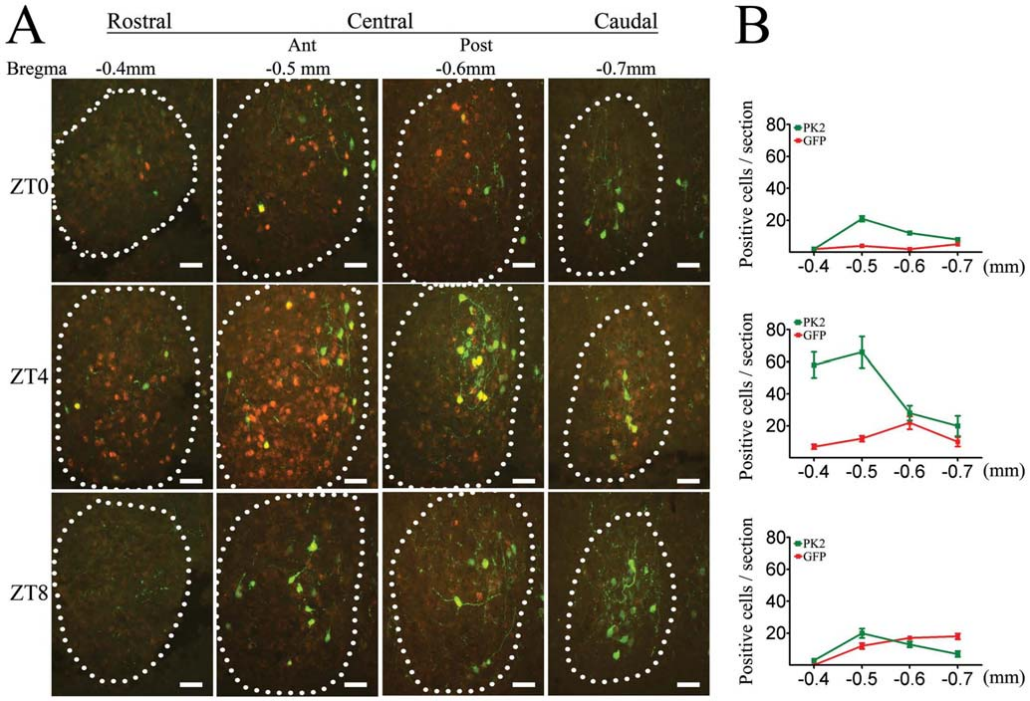
The EGFP reporter labeled a subset of *PK2* mRNA-expressing neurons in the SCN. A. On consecutive coronal sections of the SCN at ZT0, ZT4 and ZT8, fluorescence *in situ* hybridization for *PK2* mRNA (in red color) and immunofluorescence staining against EGFP protein (in green color) revealed partial overlap between the *PK2* mRNA and the EGFP reporter expressions. Most of the *PK2* mRNA-expressing cells in the caudal part of the SCN were successfully labeled by the EGFP reporter, but the majority of *PK2* mRNA-expressing cells in the rostral part of SCN did not express EGFP reporter. Scale bar  =  50 µm. B. Quantification of *PK2* mRNA-positive and EGFP protein-positive cells at different ZT time points. Error bars (SEM) were present in all data points, although some were too small to see.

**Figure 8:**
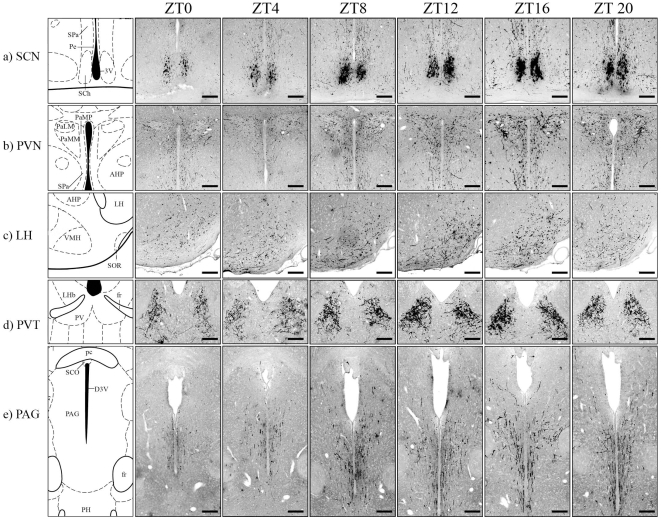
Circadian variation in the intensity of EGFP-ir fibers in many SCN target sites correlated with the expression of EGFP in the SCN. Side-by-side comparisons of coronal sections through the suprachiasmatic nucleus (SCN), paraventricular nucleus (PVN), lateral hypothalamic area (LH), paraventricular thalamic nucleus (PVT) and periaqueductal gray (PAG) during 24 hours showed that the number of EGFP-positive cells in the SCN reached its nadir at ZT0 and peaked around ZT12. Meanwhile, the intensities of EGFP-ir fibers in many SCN target areas also ebbed and flowed following the same chronological pattern. Scale bar  =  100 µm.

We also compared the expressions of endogenous *PK2* mRNA and the EGFP reporter in the SCN (Figure 7). *In situ* hybridization against the *EGFP* mRNA showed a similar chronological expression pattern as the endogenous *PK2* mRNA, reflecting the activity of the *PK2* promoter used for the transgene approach (data not shown). As for the EGFP protein, a phase delay was observed when compared with *PK2* mRNA. At ZT0, *PK2* mRNA was expressed in only a few cells, then spread throughout the whole rostral-caudal extent of the SCN at ZT4 and diminished to the minimal level after ZT8. However, the peak of the EGFP protein expression was observed around ZT12 (Figure 7A and 7B, also see Figure 8).

At ZT4 in the SCN, only slightly more than 10% of the *PK2* mRNA-expressing cells could be labeled by the EGFP reporter in the rostral and anterior central quadrants (13±4 out of 124±14 *PK2+* cells were EGFP-ir on 14 µm section, three animals). In contrast, about 60% of *PK2* mRNA-expressing cells were positive for EGFP signal in the posterior central and caudal SCN (29±7 out of 48±6 *PK2+* cells were EGFP-ir on 14 µm section, three animals, also see Figure 3 for the preferential expression of EGFP reporter in the caudal SCN on parasagittal section). On the other hand, comparable (sixty) percentage of EGFP-ir cells in both the rostral (9±4 out of 13±4 EGFP-ir cells were *PK2+* on 14 µm section, three animals) and caudal SCN (19±5 out of 29±4 EGFP-ir cells were *PK2+* on 14 µm section, three animals) expressed *PK2* mRNA at ZT4 (Figure 7). It is noteworthy that in the MPO, where the expression of *PK2* mRNA doesn’t change over time, about 78% of the *PK2* mRNA-expressing neurons were positive for EGFP reporter (47±8 out of 60±7 *PK2*+ neurons were EGFP-ir, three animals). Most importantly, all EGFP positive neurons in the MPO expressed the endogenous *PK2* mRNA (data not shown).

### Fluctuation of EGFP-ir fibers in many SCN target areas

Interestingly, we observed a wax and wane in the intensities of EGFP-ir fibers in many SCN target areas, following the same chronological pattern as the EGFP-ir neurons in the SCN (Figure 8). Side-by-side comparisons of coronal sections through the SPa, PVN, LH, PVT and PAG during 24 hours showed moderate oscillation of the amounts of EGFP-ir fibers in these areas, with the densest and strongest staining appeared around ZT12 and the lightest staining appeared around ZT0 (Figure 8). Density of the EGFP-ir fibers in the DMH, which also receive immense innervations from the MPO, did not show noticeable change during the 12L:12D cycle (data not shown).

## Discussion

Previous studies have indicated that PK2 and its receptor PKR2 are important output components of the central circadian clock in the SCN [15], [18]–[19], [21]. The rhythmic expression of PK2 in the SCN and the complementary distribution of PKR2 in most primary SCN target areas [20]–[21] are consistent with the prospective role of PK2 as an output molecule to regulate multiple circadian rhythms in the rodent [15]–[16]. In this study, we confirmed that a subset of PK2-expressing neurons in the SCN projected to many primary target areas of the SCN. Given its secreted nature, these findings suggested that PK2 would be released at the terminals of efferent projections to regulate circadian-controlled processes.

To preserve most of the transcriptional units for *PK2* gene expression, a BAC clone containing the *PK2* gene and over 200 kb flanking sequences was used for the transgenic study. Overall, the distribution of EGFP-expressing cells in the brain of transgenic mouse matched the *PK2* mRNA expression observed in previous *in situ* hybridization studies [17], [20]. There were a few exceptions, however, such as many neurons in the dorsal tenia tecta (DTT) and septohippocampal nucleus (SHi) were positive for EGFP, but no evident *PK2* mRNA expression was detected in these areas in the previous *in situ* hybridization study [20]. On the other hand, many cells in the islands of Calleja expressed *PK2* mRNA, but only a few EGFP-ir cells were observed in the major island. This inconsistency might be caused by random insertion of the transgenic construct into the chromosome, a common unintentional outcome of the transgene approach. On the other hand, insertion of the EGFP reporter cassette after the *PK2* promoter might nullify a potential enhancer in the first intron of *PK2* gene. All these could contribute to the errant expression of the reporter gene in the transgenic mouse.

One distinctive characteristic of *PK2* gene expression inside the SCN is its dramatic oscillation during the circadian cycle [15]–[17]. *In situ* hybridization against the *EGFP* mRNA in the SCN showed a similar rhythmic expression pattern, reflecting the activity of *PK2* promoter. Comparison of the dynamics of *EGFP* mRNA, *PK2* mRNA and EGFP protein expression in the SCN showed that both *EGFP* and *PK2* mRNA peaked at ZT4, although the peak of EGFP protein expression lagged 6–8 hours behind and peaked around ZT12, presumably due to some sort of delay in the protein translation process. On the other hand, when the *EGFP* and *PK2* mRNA diminished to undetectable level at night, the EGFP protein persisted in the SCN, most likely owing to the long half-life (>24 hours) of the EGFP reporter used in this transgenic mouse [26].

### EGFP reporter labeled a subset of PK2 mRNA-expressing neurons in the SCN

Using combined *in situ* hybridization against *PK2* mRNA and immunostaining for the EGFP protein on the same frozen section, we found that as many as 80% of the *PK2* mRNA-expressing neurons could be labeled by the EGFP reporter in the MPO, where the expression of *PK2* mRNA was not oscillating during circadian cycle. However, only a subset of *PK2* mRNA-expressing cells in the SCN was labeled by the EGFP reporter. In the posterior central and caudal quadrants of the SCN, the EGFP reporter was detected in more than 60% of *PK2* mRNA-expressing neurons. While in the rostral and anterior central quadrants of the SCN, EGFP reporter only labeled about 10% of *PK2* mRNA-expressing cells, mostly in the dorsomedial subregion. Taking into consideration of the phase delay between the *PK2* mRNA and EGFP protein expression, the EGFP reporter would have labeled more *PK2* mRNA-posoitive cells in this region. The transcription of *PK2* gene has been supposed to be controlled by the binding of CLOCK/BMAL1 heterodimer to several E-box elements on the promoter of *PK2* gene [15]–[16], which were preserved in the BAC cloned used in the transgene construct. However, the absence of the EGFP reporter expression in the majority of the *PK2*-expressing cells in the anterior part of the SCN suggested that the transcriptional regulation of *PK2* gene in the anterior SCN neurons might be different from the posterior SCN counterparts. CLOCK/BMAL1 and the upstream promoter of *PK2* gene would be sufficient for the activation of EGFP reporter in the posterior SCN. However, other transcription factors and a transcription enhancer, which most likely located in the first intron and was tampered by the insertion of the EGFP reporter cassette, would be essential for the authentic expression of *PK2* gene in the anterior SCN.

A long-standing hypothesis assumes two separate, but mutually coupled, circadian oscillators that drive the onset and end of activity, and respond to dawn and dusk differentially[27]–[28]. Recent evidence suggest that differential oscillatory “evening” or “morning” machineries correspond to groups of neurons in the anterior or posterior divisions of the SCN [29]–[31]. As the EGFP reporter mainly represented the subset of *PK2* mRNA-expressing cells in the caudal divisions of the SCN. It would be interesting to investigate whether these EGFP-positive neurons correlated to the “morning” cells. Further investigation on the characteristics of the EGFP-positive neurons would shed light on the role of these particular groups of neurons in the central circadian clock.

### Possible routes of PK2 in transmitting circadian information

PKR2, a G-protein coupled receptor for PK2, has been detected in major SCN target sites by *in situ* hybridization and ligand binding autoradiography [20]–[21]. In this study, we confirmed that the EGFP-labeled/*PK2*-expressing cells in the SCN projected to most SCN target areas, including the LSV, BSTM, MnPO, SPa, PVN, DMH, LH, PVT and PAG. Four different routes of projections for the EGFP-positive fibers could be traced out of the SCN. 1) The densest EGFP-ir fibers directed dorsally through the SPa and innervated the PVN; 2) Some EGFP-ir fibers turned caudally after leaving SCN and most likely went into DMH; 3) Rostrally, a few EGFP-ir fibers extended into the preoptic area, thereafter it may further extended into MnPO, BSTM and LSV; 4) Caudally, many EGFP-ir fibers appeared to target the posterior hypothalamus, especially the Arc. These findings suggested that PK2 might be axonally transported and released at the terminals, given its secreted nature, to regulate various circadian controlled processes by activating the receptor PKR2 expressed in these target sites. We observed little EGFP-ir fibers in some SCN target sites that were determined by dye-tracing and immunocytochemistry experiments, such as the anterior paraventricular thalamic nucleus and parataenial nucleus [4], [6], [9]. Considering that the EGFP reporter failed to label all *PK2* mRNA-expressing cells in the SCN, we could not rule out the possibility of the PK2-expressing/EGFP-negative neurons in the SCN also projected to these sites.

During peak expression, the *PK2*-expressing cells are scattered in both the dorsomedial and ventrolateral SCN (see Figure 7A and [15], [17], [20]), which are two functionally and morphologically distinct sub-regions that are frequently recognized as vasopressin- (AVP) or vasoactive intestinal peptide- (VIP) expressing groups [13], [32]–[34]. Immunohistochemical studies reveal AVP and VIP positive fibers originating from the SCN in all known SCN target sites within the hypothalamus, largely overlapping with each other [5], [9]. In their double-labeled *in situ* hybridization study in the rat SCN, Masumoto et al [17] found nearly identical (about 50%) co-localization of *PK2* mRNA with that of *AVP* or *VIP*. In this study, however, the vast majority of the EGFP-positive cells were located in the dorsomedial and caudal SCN, with most co-expressed with AVP and virtually none co-expressed with VIP. Even though approximately 60% EGFP-positive neurons in the SCN co-expressed AVP, there was a notable difference between the distribution of AVP-positive and EGFP-positive fibers in the target sites of SCN, particularly the limited presence of EGFP-positive fibers in the anterior paraventricular thalamic nucleus and ventromedial hypothalamic nucleus, suggesting that discrete groups of SCN neurons might have preferential projection targets.

Interestingly, we observed a circadian variation in the intensities of EGFP-ir fibers in many of the SCN target sites, such as the PVN, LH and PVT, which bore a resemblance to the oscillation of EGFP expression in the SCN during 24 hours. Dye tracing experiments reveal that these areas also receive inputs from the MPO [24], another area which exhibits a strong co-expressing of *PK2* mRNA and EGFP protein in the transgenic mouse. Nonetheless, the expression of EGFP reporter in MPO and other areas (such as OB, dorsal tenia tecta, septohippocampal nucleus, horizontal limb of the diagonal band of Broca and arcuate nucleus) did not change over the daily cycle, implying that the observed ebb and flow of EGFP-ir fibers in these areas were caused by changes of EGFP signals in the SCN. DMH, a hypothalamic nucleus involved in a variety of behavioral and physiological responses, has been considered as one of the major output targets of SCN [23]. Although there was little variation in the intensity of EGFP-ir fibers in the DMH, we confirmed that some of the EGFP-expressing neurons in the SCN indeed extended into DMH, using the retrograde tracer Fluoro-Gold.

Our study also demonstrated an innervation of the contralateral SCN, which has been reported in previous dye-tracing experiment in hamster [6]. In rat, vasoactive intestinal polypeptide (VIP) containing fibers could be observed to traverse the optic chiasm in immunostaining studies [35]. It has been suggested that the reciprocal innervation of the bilateral SCN serves to couple the two distinct circadian oscillators, which seems to correspond to the left and right sides of the bilaterally paired SCN [36]–[38]. *PKR2* mRNA-expressing neurons are clustered in the dorsolateral region of the SCN [17], which has important roles to relay or integrate the phase-resetting information to autonomously oscillating cells [39]–[40]. It is plausible that PK2-PKR2 system might play some role in integrating the circadian phases of the two paired SCN.

In summary, this study showed that PK2-expressing cells in the SCN projected into many known SCN target sites, indicating that PK2 could reach these sites through axonal transportation. Further studies are warranted to determine the role of distinct groups of *PK2*-expresing cells in the SCN and how PK2 is released at the terminals and transmits the circadian information of the central clock.

## Materials and Methods

### Transgenic animal

A PK2-EGFP transgenic mouse was generated by the GENSAT project at Rockefeller University [41]. Briefly, the bacterial artificial chromosome (BAC) clone RP23-12A18 was genetically modified so that the enhanced green fluorescence protein (EGFP) gene followed by a polyadenylation signal was inserted after the promoter of *PK2* gene (Figure 1A). The 250 kb BAC clone contained the entire transcriptional unit for *PK2* gene, with 127 kb upstream and 110 kb downstream sequences. Cryopreserved embryos (011832-UCD-Embryos) of PK2-EGFP transgenic mouse were obtained from the Mutant Mouse Regional Resource Centers (MMRRC, University of California at Davis) and recovered by the Transgenic Mouse Facility at University of California, Irvine. Presence of the transgene was determined in mouse tail genomic DNA by PCR, using primer1 (5-CCTACGGCGTGCAGTGCTTCAGC-3) and primer2 (5-CGGCGAGCTGCACGCTGCCGTCCTC-3). Homozygous transgenic mice were bred from hemizygous and used in further analysis. No developmental and behavior abnormalities were observed in the homozygous mice. Mice were housed with food and water available *ad libitum* in a light-controlled (12 h light: 12 h dark cycle; light turned on at 7AM) and temperature-controlled (22±1°C) mouse facility. All animal procedures were conducted in accordance with the Guidelines for the Institutional Animal Care and Use Committee at the University of California, Irvine.

### Perfusion and immunofluorescence staining

Eighteen mice of 8–10 weeks age, three for each time point, were sacrificed at zeitgeber time (ZT) 0, 4, 8, 12, 16 and 20. Animals sacrificed in the dark were deeply anesthetized with pentobarbital (150 mg/kg) under the dim red light illumination and their heads were completely covered to prevent light from reaching the eyes. Mice were perfused intracardially with 50 ml 1X PBS (2.7 mM KCl; 1.8 mM KH2PO4; 10.1 mM Na2HPO4; 137 mM NaCl) followed by 50 ml 4% paraformaldehyde in 1X PBS. Brains were post-fixed at 4°C for 24 hours in the same fixative, cryoprotected in 30% sucrose in 1X PBS for 24 to 48 hours. Forty-micron coronal or sagittal sections were processed free-floatingly for single- or double-label immunostaining. Primary antibodies used in this study included rabbit polyclonal anti-GFP (1:1000, Invitrogen, Carlsbad, CA), chicken polyclonal anti-GFP (1:1000, Abcam, Cambridge, MA), rabbit polyclonal anti-vasopressin (1:5000, Chemicon, Temecula, CA) and rabbit polyclonal anti-VIP (1:2500, Abcam, Cambridge, MA). Every third section was incubated with primary antibodies at 4°C overnight, and then labeled with Alexa488- or Alexa568-conjugated goat secondary antibody (1:300, Invitrogen, Carlsbad, CA). The sections were then counterstained with Hoechst 33342 (1:10000, Invitrogen, Carlsbad, CA) to label cell nuclei and mounted with Fluoromount-G (SouthernBiotech, Birmingham, AL). In each immunostaining, sections from each time point were processed together to minimize variability attributable to handling conditions.

### Fluorescence in situ hybridization and immunofluorescence staining

Fourteen-micron coronal sections were mounted onto Fisherbrand Superfrost Plus slide (Thermo Fisher Scientific, Pittsburgh, PA). The *PK2* cDNA fragment-containing vector was linearized with restriction enzyme and used as template to synthesize anti-sense complementary RNA probes, using a digoxigenin (DIG) RNA labeling mix (Roche Applied Sciences, Indianapolis, IN). Every seventh coronal section was processed simultaneously for *in situ* hybridization and immunostaining as described previously [42]. Briefly, after high-stringency post-hybridization wash, sections were incubated in hydrogen peroxide to quench endogenous peroxidase activity, washed with 1X PBS, blocked in 10% donor horse serum (Omega Scientific, Tarzana, CA) for one hour and incubated with a horse radish peroxidase-conjugated anti-DIG antibody (1:500, Perkin Elmer, Waltham, MA) at 4°C overnight. *PK2* mRNA-expressing cells were revealed with the TSA Plus Fluorescence Kit (Perkin Elmer, Waltham, MA) as described in the instruction manual. The sections were then immunostained as described on above section.

### Retrograde axonal tracing

The stereotaxic injections of Fluoro-Gold (Fluorochrome, Denver, CO) into fourteen adult male transgenic mice (33–40 g) were performed as described [43]. The coordinates of the dorsal medial hypothalamic nucleus (DMH) regions were −1.9 mm anterior-posterior, 0.3 mm medial-lateral, −5.2 mm dorsal-ventral from the bregma, according to the atlas of Franklin and Paxinos [44]. Animals were anesthetized with a mixture of ketamine (100 mg/kg) and xylazine (10 mg/kg). Fluoro-Gold was dissolved in 0.9% NaCl at 4% concentration and 0.2 microliter dye was delivered through a Hamilton microsyringe using an automatic syringe pump (KD Scientific). After a survival time of seven days, animal was deeply anesthetized with pentobarbital (150 mg/kg) and perfused intracardially between 6:30PM to 7:30PM as described above. The brain was removed, post-fixed overnight at 4°C and cryosectioned. Forty-micron sections were collected. For those animals with the Fluoro-Gold deposits restricted primarily within the borders of the DMH, immunodetection of EGFP-expressing cells within the SCN was performed using rabbit anti-GFP antibody. Fluoro-Gold labeled neurons were detected by direct fluorescence.

### Image analysis

For immunofluorescence staining and Fluoro-Gold tracing, multichannel fluorescence images of the brain sections were captured using a CCD camera attached to a Carl Zeiss Axiovision fluorescence microscope (Axiovert 200M), and exported as TIFF image files using Axiovision LE (Carl Zeiss, Germany). For DIG-labeled *in situ* hybridization, images were captured using a SPOT camera system (Diagnostic Instruments Inc., Sterling Heights, MI) attached to a Carl Zeiss Axioskop2 Plus light microscope. Adobe Photoshop was used to adjust the brightness and contrast of images, so that the background of all images appeared similar.

### Cell counts

Only cells with positive signals stronger than the background were tallied for quantification. Cell counts were performed by counting all cells within the boundaries of the SCN in each section, and total cell number in the whole SCN were estimated using the stereological method as described [9]. Statistical analysis was carried out with GraphPad Prism software.
